# Effect of High Temperature Exposure and Laboratory Processing Techniques on the Diagnostic Performance of Dry Swabs for the Detection of African Swine Fever Virus

**DOI:** 10.3390/v16121812

**Published:** 2024-11-21

**Authors:** Leonard Izzard, David T. Williams, Peter A. Durr

**Affiliations:** CSIRO, Australian Centre for Disease Preparedness (ACDP), Geelong, VIC 3220, Australia; d.williams@csiro.au (D.T.W.); peter.durr@csiro.au (P.A.D.)

**Keywords:** ASFV, swab, surveillance, heat, DSe

## Abstract

One of the key surveillance strategies for the early detection of an African swine fever (ASF) incursion into a country is the sampling of wild or feral pig populations. In Australia, the remote northern regions are considered a risk pathway for ASF incursion due to the combination of high numbers of feral pigs and their close proximity to countries where ASF is present. These regions primarily consist of isolated arid rangelands with high average environmental temperatures. A specific objective of this study was to assess whether the exposure of swabs to the high temperatures that may be encountered in outback Australia, over an extended period, would reduce the diagnostic sensitivity (DSe) of real-time PCR (qPCR) to detect ASF virus (ASFV). We found that the extended heat exposure (up to 45 °C) of FLOQSwabs or GenoTube swabs, either prior to blood sampling or post sampling, showed no reduction in the DSe of the ASFV qPCR compared to swabs stored at room temperature (~21 °C). We also assessed an improved DNA extraction method for samples collected using GenoTube swabs to obtain DSe results comparable to FLOQSwabs. Taken together, these experiments demonstrate that dry swabs can provide the basis for an effective low-cost surveillance system for ASF in situations where extended exposure to high environmental temperatures is unavoidable.

## 1. Introduction

The current Eurasian pandemic of African swine fever (ASF) began with its detection in Georgia in 2007 [1]. It then spread northwards to the Russian Federation [2] and, from there, westwards into the European Union, reaching Belgium in 2018 [3]. In the same year, it was also detected in China where it caused a massive outbreak [4] followed by rapid spread into Vietnam and other parts of Southeast Asia [5]. In 2019, ASF emerged in Timor-Leste for the first time [6] and, in 2020, outbreaks were detected in the highland provinces of Papua New Guinea [7]. ASF continues to circulate in Timor-Leste and Papua New Guinea, causing severe socio-economic impacts, and threatening neighbouring countries in the Pacific region and Australia. A novel feature of the current ASF pandemic has been the role of wild pig populations in the epidemiology of the disease [8]. Based on the experience of the eradication of the longstanding incursion into Spain (1960–1995) the disease was assessed as self-limiting in wild pig populations [9]. However, in high density populations in Estonia and Latvia, there is evidence of wild boar maintaining the disease [10,11] and it has been suggested that the virus might circulate unnoticed in wild boar populations in Asia [12].

Australia has a large feral pig population, particularly in the northern tropics [13]. Although the risk of a direct introduction of the ASF virus (ASFV) into this population is generally considered low [14], there is a risk pathway for introduction via ad hoc landings of cruising yachts and illegal fishing boats into northern Australia [15]. A particular problem if ASF were to enter this population would be its detection, as tropical Australia has a very low human population density and exotic animal disease surveillance is focused on the high-risk coastal areas where a specific surveillance programme, the Northern Quarantine Strategy (NAQS), is in operation [16]. Thus, it is not unfeasible that, were ASFV to enter the Australian feral pig population, it might circulate for an extended period before detection, by which time extensive spread may have taken place, leading to the delayed implementation of control and eradication measures. Accordingly, there is an urgent need to develop optimised systems to enable early detection, particularly using sampling techniques that can readily be deployed by land managers, indigenous rangers, stockpersons and recreational hunters.

Real-time quantitative (q)PCR is the frontline diagnostic test for ASFV in clinically affected animals, for which a preferred sample is whole blood collected with anticoagulant and ideally kept cool during transport [17]. The challenges associated with collecting whole blood and maintaining a cold chain for feral/wild pig passive surveillance, where the pigs may be found dead in remote locations, has led to the exploration of more practical sampling methods, particularly the use of dry blood. Petrov et al. [18] compared the efficacy of blood collection by three dry swab types for the detection of ASFV nucleic acid by PCR under laboratory conditions. Three dry blood swabs were evaluated: simple cotton swabs, flocked swabs with a nylon coated head (“FLOQSwab”) to facilitate elution of the specimen and a livestock genotyping swab (“GenoTube”) which preserves sample DNA. Reliable detection of ASFV nucleic acid using qPCR from whole blood collected with each of the three swab types was demonstrated. In a follow-up study, Carlson et al. [19] showed that the GenoTube swabs were suitable for the detection of ASFV nucleic acid with a high degree of accuracy from field submissions, and could also be used to detect antibodies from eluted blood samples.

These studies demonstrated that swabs have considerable potential for collecting dried blood in remote locations such as northern and outback Australia. Nevertheless, commercial swabs containing DNA preservatives have a caveat that pre sampling they should be stored at room temperatures (i.e., 18–25 °C). In practice, if these swabs are to be used in remote parts of Australia, then they may experience much higher temperatures whilst stored in the vehicles of potential personnel involved in the front-line detection of an ASF incursion. The degradation of the DNA-preservative properties of swabs may impact qPCR test sensitivity. To address the potential of commercially available dry swabs for use in active surveillance in northern Australia, we investigated the effect of high-temperature incubation and storage before and after infected blood sampling on the sensitivity of molecular diagnosis.

## 2. Materials and Methods

### 2.1. Experimental Infection of Pigs

The EDTA blood samples used in the swab experiments were collected from pigs experimentally infected with the highly virulent Georgia 2007/1 (GRG) isolate of ASFV, during two unrelated infection trials at CSIRO, Australian Centre for Disease Preparedness (ACDP). These studies were approved by the CSIRO Australian Centre for Disease Preparedness Animal Ethics Committee (AEC #1961 and #1988).

Six-week-old Landrace cross pigs were used for both infection trials and were housed for two weeks to allow for acclimatisation prior to challenge. For both trials, the pigs were anaesthetised prior to inoculation and baseline bloods and sera were collected.

For infection trial #1 (AEC #1961), six pigs were inoculated with 10^4^ TCID_50_ of GRG in 4 mL PBS via the oronasal route, as previously described [20]. For infection trial #2 (AEC #1988), eight pigs were inoculated with 10^3^ TCID_50_ of GRG in 1 mL PBS via the intramuscular route into the hind rump. The pigs were then monitored daily prior to clinical signs and twice daily following the development of clinical signs. At a predefined humane endpoint, when the animals showed moderate clinical signs over 3 consecutive days or developed severe clinical signs, the animals were sedated and humanely killed with an overdose of pentobarbitone, as previously described [20].

For infection trial #1, bloods were collected on days 1, 3, 7, 9, 11, 14 and 17 post inoculation (PI) and at clinical endpoints (between days 11 to 18 PI). For infection trial #2, bloods were collected on days 1, 3 and 5 PI. Clinical endpoint samples (days 3 to 6 PI) were collected from 7 of the 8 pigs. Following collection, tubes containing 5 mL aliquots of EDTA blood were frozen at −80 °C.

The whole blood used in experiment #1 of this study was collected from infection trial #1, while the whole blood used in experiments #2 and #3 was collected from infection trial #2. All blood samples were pre-tested for the presence of ASFV via qPCR. All blood samples from infection trial #1 were used for experiment #1, whereas only ASFV-positive bloods from infection trial #2 were used for experiment #2 and #3.

### 2.2. Swabs

Two commercial dry swab products, GenoTube Livestock Swabs (Thermo Fisher Scientific, Scoresby, Melbourne, Australia) and Human DNA free FLOQSwab (COPAN Diagnostics Inc., Murrieta, CA, USA) were assessed. Both swabs have DNA-preservative properties. The manufacturer’s recommended storage temperature for the FLOQSwab is 2–30 °C, while for the GenoTube recommended storage it is between 15 and 30 °C.

### 2.3. Swab Experiments

Three inter-related experiments were undertaken to assess the effect of high temperatures on swabs, both pre sampling and post sampling exposure, as well as to optimise the extraction protocol for GenoTube swabs.

#### 2.3.1. Experiment 1: Swab Pre-Sampling Temperature Treatment

This experiment was designed to assess the impact of high temperature exposure on the selected commercial swabs prior to blood sampling. Prior to blood sampling, the two types of commercial swabs (FLOQSwab and GenoTube) were divided into four treatment groups each. Two groups from each swab type were incubated at 37 °C for a period of either 8 or 38 weeks, while the remaining two groups from each swab type were incubated at 45 °C for the same time periods. A separate set of control swabs were incubated at room temperature (~21 °C) for 8 weeks.

Following this high temperature pre-treatment, swabs from each treatment group were used to sample a total of 14 different blood samples from the experimentally infected pigs from infection trial #1 (Table 1) in triplicate, thus resulting in 30 swabs per blood sample (2 swab types × 5 treatment groups × 3 technical replicates) and 420 swabs in total. The swabs were then stored at room temperature for 24 hrs to dry and to simulate the delay between field sampling and arrival at the laboratory. The swab head was then cut off and added to 1 mL of virus transport media (PBS containing 1% glucose, 0.0005% Phenol Red, 1% Foetal Bovine Serum, 800 IU/mL Penicillin, 800 µg/mL Streptomycin, 6 µg/mL Amphotericin B and 0.05 mg/mL Gentamicin), then incubated on a plate shaker at 37 °C for 30 min. A 120 µL aliquot was then added to 315 µL of MagMax Lysis/Binding Solution (Thermo Fisher Scientific) and nucleic acid was extracted from 180 µL of lysate using the MagMAX™ Pathogen RNA/DNA Kit (Thermo Fisher Scientific) using a KingFisher™ Purification System (Thermo Fisher Scientific), following the manufacturer’s instructions.

#### 2.3.2. Experiment 2: GenoTube Swab Processing Protocol Comparison

This experiment was designed to assess the effect of different GenoTube swab processing protocols on the sensitivity of ASFV qPCR testing. This involved comparing the complete protocol, including different extraction/elution buffers, as well as different buffer volumes (dilution factors).

Triplicate GenoTube swabs were dipped into 21 blood samples collected from infection trial #2, then stored for 24 hrs at room temperature (63 swabs in total). Duplicate ~0.5 cm^2^ pieces of dried blood swab were excised from each swab using sterile scissors and processed using two different protocols (126 samples) (Table 1), as follows.

The first (PBS) protocol was adapted from a previously published method [19]. In brief, one of the two swab pieces was added to a 2 mL microcentrifuge tube containing silicon-carbide shards (Daintree Scientific Australia, St Helens, Australia) in 400 µL of sterile PBS and then macerated for 20 sec at 6.5 m/s in a FastPrep^®^-24 homogenizer (MP Biomedicals, Santa Ana, CA, USA). Following this, 120 µL of homogenate was added to 315 µL of MagMax Lysis/Binding Solution (Thermo Fisher Scientific) and nucleic acid was extracted as described above.

The second (Lysis buffer) protocol was adapted from an alternative method supplied by the manufacturer. In brief, a swab piece was added to a 2 mL microcentrifuge tube containing 400 µL of MagMax Lysis/Binding Solution (Thermo Fisher Scientific) and mixed using a benchtop vortex mixer (Benchmark Scientific Inc., Sayreville, NJ, USA) at top speed for 5 sec. Following this, 180 µL of lysate was used for nucleic acid extraction.

As the blood samples used in this experiment were different to those used in experiment #1, direct comparison between the two was not possible. Therefore, a control group (VTM) was prepared by dipping an additional set of GenoTube swabs into the 21 blood samples from infection trial #2, then processing as per experiment #1 (63 additional samples).

#### 2.3.3. Experiment 3: Swab Post-Sampling Temperature Treatment

This experiment was designed to assess the effect of high temperature exposure following blood sampling on the sensitivity of ASFV DNA detection. Six replicate swabs from each swab type were dipped into blood collected from 7 clinical endpoint samples from infection trial #2 (84 swabs in total) (Table 1). Three replicates from each group were then incubated at 45 °C for 7 days. The remaining 3 were stored at room temperature (21 °C) for 24 hrs. FLOQSwabs were then processed as per the method described in experiment #1, while GenoTubes were processed using the ‘Lysis buffer’ method described in experiment #2.

### 2.4. Real-Time PCR Detection and Copy Number Estimation

Real-time qPCR was performed using a modification of the World Organisation for Animal Health-recommended King assay [21]. Briefly, the assay contained sense and antisense primers (300 nM), TaqMan probe (250 nM), AgPath-ID one-step RT-PCR reagents and 5 µL of template in a 15 µL final volume. Cycling conditions were 45 °C for 10 min, 95 °C for 10 min followed by 45 cycles of 95 °C for 15 s and 60 °C for 45 s. Gene copy numbers (herein referred to as ‘copy numbers’) were determined using a standard curve by running a log dilution of a synthetic target (gBlock) alongside the samples.

### 2.5. Statistical Analyses

The three studies were treated as independent experiments, each being analysed by an analysis of variance (ANOVA) within the generalised linear modelling framework [22]. The response variable in all cases was the calculated copy number per ml, although to assist with the homogeneity of variance required for ANOVA analysis, this was transformed by a log_10_ calculation. For experiment #1, a two-way factorial ANOVA was used with the treatment factors being the five temperature and storage time combinations (i.e., 21 °C for 8 weeks, 37 °C for 8 and 38 weeks and 45 °C for 8 and 38 weeks), to each of which the two swab types (i.e., GenoTubes and FLOQSwabs) were applied. For experiment #2, a one-way ANOVA was used with the treatment factors being the three swab resuspension methods (Lysis buffer, PBS, or VTM), with Tukey’s test being applied for a post hoc analysis of overall significant treatment effects. For experiment #3, a two-way factorial ANOVA was used with the treatments being the combination of temperature and a delay in extraction (i.e., 24 h at room temperature vs. 7 days at 45 °C) for both swab types.

As the aim of this study was to compare the effect of sampling variables such as temperature exposure and swab type on the analytical and diagnostic sensitivity of downstream ASFV qPCR, only samples that were positive for ASFV by qPCR were analysed.

All analyses were undertaken in R version 4.40.0, using the “aov” function of the statistics library for the ANOVAs, with post hoc comparison of overall significant treatments being conducted using the “TukeyHSD” function. Assessment of the statistical significance of the effects of the variables and factor levels was set at the standard threshold of *p* < 0.05. The boxplots plots for the three experiments were produced using the ggplot2 library.

## 3. Results

### 3.1. Experiment 1: Swab Pre-Sampling Temperature Treatment

To assess the effect of high temperature exposure on the swabs prior to blood sampling, 420 FLOQSwabs and GenoTubes were dipped in blood collected from the six pigs from infection trial #1. Of these blood samples, 210 were ASFV-positive (Table 1). The majority of these positive swabs were collected at clinical endpoints, at which all six infected pigs were positive. By contrast, all the swabs collected on day 3 were negative, except for one FLOQSwab, which was positive (Ct 28.8). Of the two pigs that were sampled on day 7, one was positive and the other negative.

Of the 210 swabs collected from ASFV-positive blood samples (Table 1), the overall median Ct was 24.90, with an inter-quartile range (IQR) of 22.71 and 27.27, when all Ct values were used for calculations. For the corresponding log_10_ copy number/mL the overall median was 6.74 (IQR: 6.02, 7.41). Equivalent median values were obtained from the average of three technical replicates for each sample, with a median Ct of 24.90 (IQR: 22.66, 27.10) and a corresponding log_10_ copy number/mL of 6.74 (IQR: 6.07, 7.43). Thus, the median values of the averaged technical replicates were used for all subsequent analyses of the five temperature x swab type x storage time treatments.

**Figure 1:**
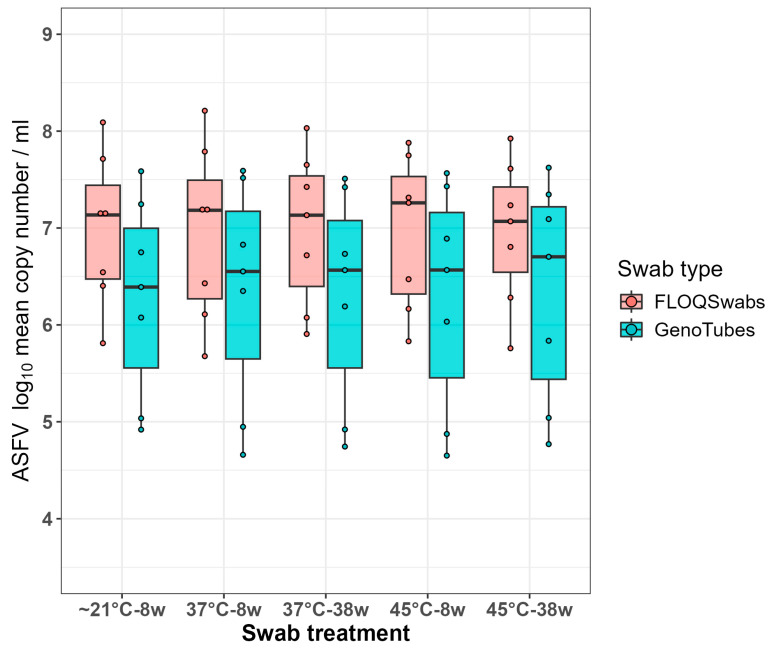
Boxplots comparing the effect of five different temperature and storage time treatments on the ASFV log_10_ copy number per mL for the two swab types, FLOQSwabs and GenoTube. Each point on the plot corresponds to the average copy number of three technical replicates.

There was overall little variation between the temperature vs. storage time for each of the two swab types, but a marked difference between the two swab types, with a median overall log_10_ copy number/mL of 7.13 for the FLOQSwabs and 6.57 for the GenoTubes (Table A1; Figure 1). As assessed by the ANOVA, this effect of swab type was statistically significant (*p* = 0.007). This analysis also confirmed that the difference was consistent across the various treatment groups, as demonstrated by a non-significant interaction (*p* = >0.999) between the groups within each swab type.

Diagnostically, these results were interpreted as FLOQSwabs showing a higher analytical sensitivity compared to the GenoTubes, but, as all the ASFV-infected samples had Ct values below the threshold of positivity for ASFV (Ct < 40), the two swab types had equal diagnostic sensitivity.

### 3.2. Experiment 2: GenoTube Swab Processing Protocol Comparison

Given the comparatively lower level of analytical specificity observed for GenoTubes in experiment #1, we evaluated the use of alternative resuspension protocols to attempt to improve sensitivity levels.

Of the 189 GenoTubes that were dipped in blood collected from the eight pigs from infection trial #2, 180 were qPCR positive (Table 1). The nine that were negative all came from one pig which was sampled on day 3 post infection. Of the 180 positive swabs, the overall median Ct value was 23.96 (IQR: 22.57, 26.65) which equated to a median log_10_ copy number/mL of 6.96 (IQR: 6.21, 7.45). For the dataset in which the three technical means were averaged (*n* = 60), the comparable median Ct value was 23.95 (IQR: 22.58, 26.60) and the median log10 copy number/mL was 6.93 (IQR: 6.18, 7.45).

**Figure 2:**
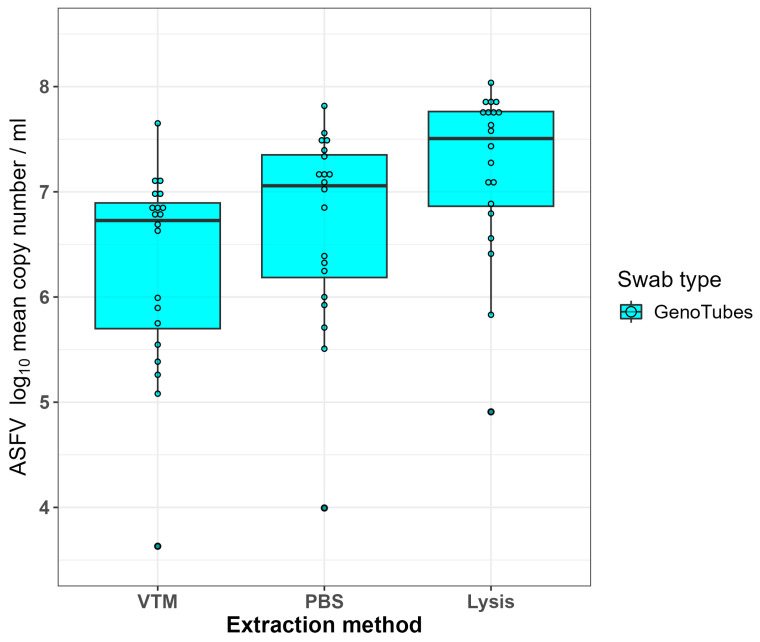
Boxplots comparing the ASFV log_10_ copy number per mL for the three different methods of extracting DNA from GenoTube swabs. Each point on the plot corresponds to the average copy number of three technical replicates.

Comparing the effect of the three different swab resuspension methods, the median Ct values were 24.61, 23.86 and 22.38 for the PBS, Lysis buffer and the VTM control, respectively (Table A2). The comparable median log_10_ copy number/mL for the three extraction methods were 6.73, 7.06 and 7.51 (Table A2; Figure 2). The log_10_ copy number obtained with the Lysis buffer resuspension method was significantly higher than the VTM control (adjusted *p* = 0.006), but the log_10_ copy number obtained using the PBS extraction method was not significantly different from either the Lysis method or the VTM control (adjusted *p*-values 0.164 and 0.354, respectively).

Overall, it was concluded that the Lysis buffer resuspension method for DNA extraction from GenoTubes was the superior method on account of the higher copy numbers obtained relative to the other two methods. However, given that the diagnostic sensitivities obtained using all three methods were equivalent, each is considered valid for diagnosing the presence of ASFV in blood.

### 3.3. Experiment 3: Swab Post-Sampling Temperature Treatment

We next assessed the effect of high temperature exposure on blood sampled with FLOQSwabs and GenoTubes on the sensitivity of detection of ASFV DNA. Based on the results from Experiment #2, the Lysis buffer resuspension method was used for processing GenoTubes swabs.

Of the 84 FLOQSwabs and GenoTubes that were dipped in blood collected from seven clinical endpoint samples from infection trial #2, all were positive (Table 1). The overall median Ct values for these swabs was 20.90 (IQR: 20.00, 21.90), which equated to a median log_10_ copy number/mL of 7.96 (IQR: 7.65, 8.23). For the dataset in which the three technical means were averaged (*n* = 28) the comparable median Ct value was 20.97 (IQR: 20.06, 21.83) and the median log_10_ copy number/mL was 7.94 (IQR: 7.67, 8.21).

**Figure 3:**
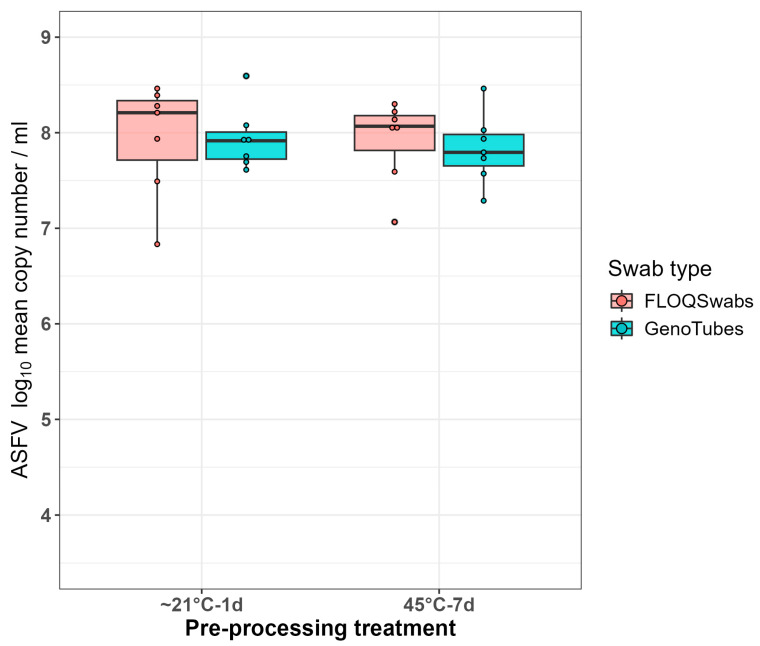
Boxplots comparing the ASFV log_10_ copy number per mL for the two different pre-processing treatments, 24 h at room temperature and 7 days at 45 °C. Each point on the plot corresponds to the average copy number of three technical replicates.

Assessing the impact on the ASFV log_10_ copy number/mL extracted from the swabs with a 7-day delay at a storage temperature of 45 °C as compared to a 1-day delay at room temperature storage; irrespective of swab type, the overall combined medians were 7.98 and 7.94 log_10_ copy numbers/mL, respectively (Table A3, Figure 3). Comparing the two swab types, the median log_10_ copy number/mL extracted from the FLOQSwabs was slightly higher than that extracted from the GenoTubes (8.10 vs. 7.86), but this was not statistically significant (*p* = 0.787).

It was therefore concluded that there was no effect of a potential extended high temperature exposure post sampling on the diagnostic sensitivity for the detection of ASFV for either type of swab.

## 4. Discussion

By means of three inter-related studies, we confirm that DNA preserving dry blood swabs are potentially an effective sampling method for the detection of ASFV in feral pigs in remote areas of Australia. Given previous work in northern Europe showing that dry swabs are effective for the detection of ASFV (and antibodies to the virus) these results are not surprising [18,19]. However, since extremes of high temperatures can be encountered in tropical and outback Australia, our work extends the environmental conditions in which this specimen collection methodology can potentially be applied. These parts of Australia are among the most difficult areas of the country in which to undertake feral pig surveillance. This is on account of the extensive geographic area that feral pig populations inhabit, the low (human) population density and the large number of feral animals, many of which are highly mobile, all factors which makes systematic, active surveillance extremely challenging. This contrasts with the European situation, where although the total area and wild pig population is comparable, the large number of hunters and a better definition of wild pig population density facilitate ASF surveillance [23]. Thus, many of the recommendations for the surveillance and management of ASF in wild boar in European situation are not particularly relevant to that of feral pigs in remote Australia and thus novel solutions are required [24].

In developing a fit-for-purpose early detection surveillance system for feral pigs in outback Australia, the first option we explored was Whatman 3 mm filter paper. This was on account of this sample collection method already having been shown to be a practical method for transporting dried blood for ASFV diagnosis under tropical conditions [25]. Nevertheless, the recounted experience of veterinarians in Australia using filter paper for blood collection was that it was cumbersome when dealing with recently shot feral pigs due to the difficulty aligning a flowing blood drop with the filter paper (Dr J. Schmidt, pers. comm.). Furthermore, in the case of a found-dead feral pig suspected to be infected with ASFV, there would be a need to cut into the carcass to create a blood flow with the danger of cut-injuries and close contact with carcass tissues and fluids. This poses a zoonotic risk, especially from brucellosis and leptospirosis, both diseases having been shown to be endemic in Australia’s feral pigs [26]. Thus, one benefit of the dry swabs is that they can readily sample recently shot animals by swabbing for blood pooled in the bullet hole and thereby avoiding opening the carcass.

Due to the advantages of ease of use, low cost and minimal biosafety risk, dry swabs can be recommended as an alternative specimen collection method for the remote detection of ASFV; however, it is important to note that it is not the optimal sampling. Pikalo et al. [27] presented data quantifying ASFV from different studies and showed that ~2-times higher log_10_ copy numbers were obtained from EDTA blood as compared to GenoTubes. Therefore, in a situation where a quality sample can be obtained by a veterinarian, the recommended sampling method for ASFV detection should then be followed. In the specific case of Australia, the current advice is to collect blood (both EDTA and plain tube) from living animals and fresh tissue from the spleen, lymph nodes, kidney, lung and ileum post mortem [28].

Another consideration for sample collection is where there is a need to isolate live virus for further characterisation and quality sequencing. Although our experiment #3 showed that a temperature of 45 °C did not impact the detection of ASFV DNA, there is limited published information on the impact of high temperatures on ASFV viability in blood or serum. Plowright and Parker [29] undertook virus inactivation experiments under various conditions and found that, although a medium containing ASFV remained infectious for 17–22 days at 37 °C, at 60 °C, ASFV in serum-containing medium lost its infectivity after only 25–30 min. Although this result cannot be directly extrapolated to a virus collected using DNA preserving swabs, the exposure of a swab sample to high temperatures following collection may inactivate the virus present in the sample. If live virus is required, then sampling with virus transport media (VTM) and cold-chain transport is recommended.

This work completes the laboratory stage of assessing the feasibility of using dry blood swabs, but it is important to note that further assessment might be required to determine the acceptability of the sampling method under field conditions. In making this caveat, it should be noted that previous attempts to recruit landowners and other potential samplers (like stockpersons and recreational hunters) to take samples from feral pigs have not always been successful. An example of this was a study undertaken in the rangelands of outback NSW which aimed to recruit recreational hunters to collect serum samples from killed feral pigs for the early detection of FMD [30]. Despite the study being well planned and resourced, there was a reluctance by hunters to participate, with less than 0.5% of the estimated population of pig hunters inquiring about the programme. There was also a low rate of return of sera from hunters who had been sent the sampling kit, with 18% of a potential total of 1600 blood samples returned for analysis. The reasons for this poor uptake were diverse, but one important factor was the relative complexity of the sampling regime on account of the need to avoid haemolysis, requiring that the participants manually separate the sera from the clot and keep the serum cool. As the protocol for collecting blood and other fluids for ASF surveillance using dry swabs is much more straightforward, we would expect there to be fewer problems with the recruitment of land managers. Nevertheless, a formal stakeholder engagement exercise to determine the acceptability of using the dry swabs might be considered along with the development of printed and video material on the sampling protocol. However, as Australia is currently free of ASFV, the availability of fresh samples would be limited. So, a possible future direction could be to undertake field sampling within our neighbouring Southeast Asian regions where the agent is currently circulating.

## Figures and Tables

**Table 1:** Tally of swabs processed and analysed for the three inter-related experiments comparing storage and processing treatments for the two types of swabs.

Experiment Number	Number of Pigs	Number of Collection Points	Number of Swab Types	Number of Technical Replicates	Number of Different Treatments	TOTAL Swabs	TOTAL Positive Swabs
1	6	2 or 3 ^a^	2	3	5	**420**	**210**
2	8	1, 2 or 3 ^b^	1	3	3	**189**	**180**
3	7	1 ^c^	2	3	2	**84**	**84**

^a.^ Two pigs had blood collected at three time points and four pigs had blood collected at two time points; ^b.^ six pigs had blood collected at three time points and four pigs had blood collected at two time points; ^c.^ a single collection time point corresponded to the clinical endpoint bleed.

## Data Availability

The original contributions presented in the study are included in the supplementary material, further inquiries can be directed to the corresponding author.

## Supplementary Materials

The following supporting information can be downloaded at: https://www.mdpi.com/article/10.3390/v16121812/s1, Table S1: Experiment #1—raw; Table S2: Experiment #2—raw; Table S3: Experiment #3—raw.

## Appendix A

**Table A1:** Cross-tabulation of the technical replicate-averaged Ct values and log_10_ copy numbers/mL for Experiment #1, wherein two swab types and five storage period and temperature treatments were compared.

Swab Storage Temperature	Storage Time (Weeks)	Median (and IQR) of the Averaged Ct Value		Median (and IQR) of the Averaged log_10_ Copy Number/mL	
		FLOQSwabs	GenoTubes	FLOQSwabs	GenoTubes
RT (~21 °C)	8	23.61 (22.6, 25.79)	26.06 (24.06, 28.81)	7.13 (6.47, 7.44)	6.39 (5.56, 7)
37 °C	8	23.45 (22.42, 26.46)	25.53 (23.48, 28.5)	7.18 (6.27, 7.49)	6.55 (5.65, 7.17)
37 °C	38	23.61 (22.28, 26.04)	25.48 (23.8, 28.81)	7.13 (6.4, 7.54)	6.57 (5.56, 7.08)
45 °C	8	23.19 (22.3, 26.3)	25.48 (23.52, 29.14)	7.26 (6.32, 7.53)	6.57 (5.45, 7.16)
45 °C	38	23.82 (22.66, 25.55)	25.03 (23.33, 29.19)	7.07 (6.54, 7.42)	6.70 (5.44, 7.22)

**Table A2:** Cross-tabulation of the technical replicate-averaged Ct values and log_10_ copy numbers/mL for experiment #2, wherein two GenoTube swab resuspension methods plus a VTM control method were compared. All experiments were carried out at room temperature (~21 °C).

Swab Resuspension Method	Median (and IQR) of the Averaged Ct Value	Median (and IQR) of the Averaged log_10_ Copy Number/mL
**PBS**	24.61 (23.85, 27.86)	6.73 (5.70, 6.89)
**Lysis buffer**	23.86 (22.89, 26.73)	7.06 (6.19, 7.35)
**VTM (control)**	22.38 (21.54, 24.5)	7.51 (6.86, 7.76)

**Table A3:** Cross-tabulation of the median and IQR for the technical replicate-averaged Ct values and log_10_ copy numberers/mL for experiment #3, wherein two post-sampling storage treatments for two swab types were compared. Note that room temperature (RT) corresponds to approximately 21 °C.

Swab Storage Temperature	Median (and IQR) of the Averaged Ct Value		Median (and IQR) of the Averaged log_10_ Copy Number/mL	
	FLOQSwabs	GenoTubes	FLOQSwabs	GenoTubes
24 h at RT	20.07 (19.65, 21.7)	21.03 (20.73, 21.67)	8.21 (7.71, 8.34)	7.92 (7.72, 8.01)
7 days at 45 °C	20.53 (20.17, 21.37)	21.43 (20.82, 21.9)	8.07 (7.81, 8.18)	7.79 (7.65, 7.98)
